# Laryngeal Stridor

**Published:** 1898-04-02

**Authors:** 


### LARYNGEAL STRIDOR.

Dr. Eustace Smith some time ago expressed the opinion that laryngeal stridor, like many other nervous phenomena in early life, was sometimes due to the irritation of adenoid growths and might be successfully treated by their removal. He now records the case of an infant one month old in whom the breathing was so laboured as to raise fears for the child's life. At times the breathing would become excessively stridulous, the face livid, and the chest walls drawn in. In its ordinary state, although crowing loudly with each breath, the child did not seem to suffer discomfort. On examination adenoids were found. When about four months old, as no improvement had taken place, the adenoid vegetations were scraped away under chloroform. The child quickly recovered, and 16 days after the operation was discharged cured. Dr. Lambert Lack, however, disputes the view held by Dr. Eustace Smith, maintaining that congenital laryngeal stridor depends upon a congenital deformity of the superior laryngeal opening aided by the flaccidity of the parts in infancy, and considers that Dr. Smith's case was probably caused by spasm set up by the adenoids, this being a different thing from what is described as congenital laryngeal stridor.

